# Phase Ib study of irinotecan and ramucirumab for advanced gastric cancer previously treated with fluoropyrimidine with/without platinum and taxane

**DOI:** 10.1007/s00280-018-3678-5

**Published:** 2018-08-30

**Authors:** Hironaga Satake, Tamotsu Sagawa, Koshi Fujikawa, Yukimasa Hatachi, Hisateru Yasui, Masahito Kotaka, Takeshi Kato, Akihito Tsuji

**Affiliations:** 1 Cancer Treatment Center, Kansai Medical University Hospital, Hirakata, Osaka Japan; 2 Department of Medical Oncology, Kobe City Medical Center General Hospital, Kobe, Hyogo Japan; 3 Division of Medical Oncology, National Hospital Organization, Hokkaido Cancer Center, Sapporo, Hokkaido Japan; 4 Gastrointestinal Cancer Center, Sano Hospital, Kobe, Hyogo Japan; 5 Department of Surgery, National Hospital Organization, Osaka National Hospital, Osaka, Osaka Japan; 6 Department of Clinical Oncology, Kagawa University Hospital, Miki, Kagawa Japan

**Keywords:** Stomach neoplasms/DT, Salvage therapy/MT, Vascular endothelial growth factor receptor/AI, Antagonists and inhibitors: topoisomerase I inhibitors

## Abstract

**Purpose:**

Optimal salvage chemotherapy for patients with treated advanced/metastatic gastric cancer (AGC) is unknown. Irinotecan is commonly used in Japan. Ramucirumab, a human IgG-1 monoclonal antibody targeting the extracellular domain of VEGF receptor 2, is the first molecularly targeted agent proven to be effective in second-line therapy for AGC in combination with chemotherapy. We sought to determine the maximum tolerated dose (MTD) and recommended dose (RD) of ramucirumab plus irinotecan for AGC previously treated with fluoropyrimidine with/without platinum and taxane.

**Methods:**

Patients received systemic chemotherapy with ramucirumab (8 mg/kg) and irinotecan on day 1, repeated every 2 weeks. A decrease in irinotecan dose was planned from start level 1 (irinotecan 150 mg/m^2^). This trial was registered with the University Hospital Medical Network (UMIN no. 000018606).

**Results:**

Six patients were enrolled from August 2015 to September 2017. No dose-limiting toxicity (DLT) was observed, and the maximum tolerated dose (MTD) was not reached at level 1. Irinotecan 150 mg/m^2^ in combination with ramucirumab 8 mg/kg was administered with acceptable toxicity, and all patients were treated at these doses. No treatment-related deaths were observed. Adverse events of Grade 3/4 were neutropenia (17%), anemia (17%) and hypertension (17%). Patients were evaluated using the RECIST criteria, and response rate and disease control rate were 17% and 83%, respectively.

**Conclusions:**

Salvage chemotherapy with irinotecan plus ramucirumab was well-tolerated by patients previously treated for AGC. RD was defined as irinotecan 150 mg/m^2^ in combination with ramucirumab 8 mg/kg.

## Introduction

Gastric cancer is one of the most common malignancies and the third leading cause of cancer mortality worldwide [1]. Most of inoperable cases remain incurable, and median overall survival (OS) is only 11–14 months, even for patients who receive systemic chemotherapy [2–4].

Standard treatment for advanced/metastatic gastric cancer (AGC) consists of systemic chemotherapy. The combination of fluoropyrimidine and platinum with or without epirubicin or docetaxel is used worldwide for first-line treatment [2, 3, 5, 6]. Patients intolerant of first-line chemotherapy for AGC, or with resistant disease, have a poor prognosis. In these cases, taxanes or irinotecan are the two main options for second-line chemotherapy. A phase III study of second-line therapy that compared paclitaxel with irinotecan, the WJOG 4007 trial, showed better survival benefit in the paclitaxel group (median OS, 9.5 months in the paclitaxel arm and 8.4 months in the irinotecan arm) [7]. Ramucirumab, a human IgG-1 monoclonal antibody that targets the extracellular domain of vascular endothelial growth factor (VEGF) receptor 2, is the first molecularly targeted agent proven to be effective in second-line therapy for AGC [8, 9]. The RAINBOW phase III study compared ramucirumab plus paclitaxel with placebo plus paclitaxel, and showed the combination of ramucirumab plus paclitaxel significantly increased OS compared with placebo plus paclitaxel for AGC patients [hazard ratio (HR) 0.807 (95% confidence interval 0.678–0.962); *P* = 0.017] [9].

Paclitaxel with ramucirumab is usually selected for second-line treatment of AGC in Japan. Although evidence for the efficacy of third-line chemotherapy in AGC is limited, irinotecan is one of the most frequently selected anticancer drugs for salvage chemotherapy for previously heavily treated AGC. Combination chemotherapy with ramucirumab plus irinotecan/5-FU/leucovorin (FOLFIRI) is recognized as one of the most promising regimens for metastatic colorectal cancer [10]. To date, however, use of ramucirumab plus irinotecan for AGC patients has not been investigated, and a recommended dose (RD) of ramucirumab plus irinotecan for patients with AGC has not been established. The aim of this study was to determine the maximum tolerated dose (MTD) and RD for systemic chemotherapy with ramucirumab plus irinotecan for AGC previously treated with one or more prior chemotherapy regimens involving both fluoropyrimidine with/without platinum and taxanes.

## Patients and methods

### Inclusion and exclusion criteria

Inclusion criteria were age ≥ 20 years; histologically confirmed unresectable or recurrent gastric or gastro-esophageal junction adenocarcinoma, previously treated with one or more chemotherapy regimens involving both fluoropyrimidine and taxanes with/without platinum; evaluable lesion according to the Response Evaluation Criteria In Solid Tumors (RECIST) version 1.1; Eastern Cooperative Oncology Group (ECOG) performance status 0 or 1; estimated life expectancy ≥ 3 months; and adequate organ function, as defined by hemoglobin (Hb) ≥ 8 g/dL, absolute neutrophil count (ANC) ≥ 1500/mm^3^, platelet count ≥ 100,000/mm^3^, total bilirubin ≤ 1.5 mg/dL, serum transaminase level ≤ 150 U/L, creatinine ≤ 2.0 mg/dL, and ≤ National Cancer Institute Common Terminology Criteria for Adverse Events (CTCAE) grade 3 proteinuria. Exclusion criteria were brain metastasis, poorly controlled hypertension; any arterial thrombotic or thromboembolic events within 3 months before enrollment; > CTCAE grade 3 proteinuria; a grade 3–4 bleeding event; a history of bowel perforation; contraindication to irinotecan or ramucirumab, prior history of irinotecan administration; and synchronous or previous malignancy other than carcinoma in situ. We excluded homozygosity for UGT1A1*28 (*28/*28), UGT1A1*6 (*6/*6) and heterozygosity for both UGT1A1*28 and *6 (*28/*6) in this study. The UGT1A1*28 and *6 genotype is associated with irinotecan-induced hematologic toxicity, diarrhea, or both [11]. UGT1A1*28/*28, *6/*6 and *28/*6 are associated with severe irinotecan-related neutropenia in Japanese patients [12, 13]; in addition, the association between UGT1A1*6/*6 and severe neutropenia in Asian populations has been verified in a meta-analysis [14]. For patients with UGT1A1*28/*28 or *6/*6, the MTD of irinotecan is considered to be 150 mg/m^2^ [15, 16], and recently published guidelines recommended that *UGT1A1* phenotyping should be carried out in patients with a suspicion of *UGT1A1* deficiency, as reflected by low conjugated bilirubin, and in patients receiving an irinotecan dose of > 180 mg/m^2^ per administration [17].

This trial was carried out in accordance with the Helsinki Declaration and was approved by the ethics committee at Kobe City Medical Center General Hospital and National Hospital Organization Hokkaido Cancer Center. All patients were required to give written informed consent before entering the study.

### Study design and treatment

Protocol treatment was defined as chemotherapy consisting of ramucirumab and irinotecan. Specifically, the treatment regimen consisted of a 1-h administration of ramucirumab and 1-h administration of irinotecan on day 1, repeated every 2 weeks.

The study was designed to evaluate the maximum tolerated dose (MTD) of combination therapy with irinotecan and ramucirumab as a salvage treatment in patients with AGC, and to determine the recommended dose (RD).

Six patients were treated at dose level 1 (irinotecan 150 mg/m^2^ and ramucirumab 8 mg/kg). If ≥ 50% of the patients experienced a dose-limiting toxicity (DLT), six additional patients would be accrued at the next lower dose level (level 0; irinotecan 120 mg/m^2^) (Table 1). The MTD was defined as the dose at which ≥ 50% of the six patients experienced DLT. Treatment was repeated until disease progression, unacceptable toxicity, or withdrawal of consent. Irinotecan was delayed if, on the planned day of treatment, lab results included any of the following: ANC < 1200/mm^3^, platelets < 75,000/mm^3^, Hb < 8 g/dL, serum transaminase > 150 U/L, total bilirubin > 1.5 mg/dL, or if symptomatic toxicity occurred. Ramucirumab was delayed if, on the planned day of treatment, lab results included any of the following: ANC < 1,000/mm^3^, platelets < 75,000/mm^3^, or CTCAE grade > 3 proteinuria. The RD was defined as one dose level below the MTD. If the MTD was not achieved, even at level 1, it was regarded as the RD. DLT was defined by any of the following adverse events occurring in the first cycle: (1) Grade 4 neutropenia lasting > 7 days; (2) Grade 4 thrombocytopenia (< 25,000/mm^3^); (3) febrile neutropenia; (4) Grade 4 hypertension; (5) Grade 3 or 4 non-hematological adverse effects; (6) treatment discontinuation due to adverse events; (7) delay in starting the second cycle for > 14 days; or (8) treatment-related death. In the event of Grade 4 non-hematologic toxicities, treatment was interrupted. Prophylactic use of granulocyte colony-stimulating factor (G-CSF) was not allowed.

**Table 1:** Planned dose at each level

	Level 1	Level 0	Level − 1
Irinotecan (mg/m^2^)	150	120	90
Ramucirumab (mg/kg)	8		

### Study assessment

Pretreatment evaluation included a medical history; physical examination; complete blood cell count and serum chemistry tests; and chest, abdominal, and pelvic computed tomography (CT) scans. Clinical examination and biochemical tests were required before and during every cycle. All images for tumor responses were evaluated according to the RECIST version 1.1 [18]. All adverse events during chemotherapy were evaluated using the National Cancer Institute Common Terminology Criteria for Adverse Events (CTCAE version 4.0).

### Endpoints and statistical analysis

The primary endpoint in this study was the MTD and RD of the ramucirumab plus irinotecan regimen.

Secondary endpoints included toxicities, response rate (RR), progression-free survival (PFS) and overall survival (OS). Safety and efficacy analyses were both conducted in an intention-to-treat (ITT) population, defined as all patients enrolled in the study who received at least one dose of chemotherapy. All statistical analyses were conducted using the SPSS software package (SPSS 22.0 Inc., Chicago, IL).

This trial was registered with the University Hospital Medical Information Network (UMIN no. 000018606).

## Results

### Patients

From August 2015 to September 2017, six patients were enrolled. Characteristics of the enrolled patients are listed in Table 2. Median age was 68 years. All had gastric adenocarcinoma with intestinal-type disease, half having undergone primary resection, and all having received prior chemotherapy with fluoropyrimidine and taxane. One patient who had not received prior chemotherapy with platinum had relapsed during adjuvant chemotherapy with S-1 and had received ramucirumab plus paclitaxel as a second-line treatment before enrollment. Two patients were human epidermal growth factor receptor type 2 (HER2)-positive, and had received combination chemotherapy consisting of trastuzumab, fluoropyrimidine, and platinum as a first-line treatment. One patient had received triplet combination chemotherapy consisting of docetaxel, cisplatin, and S-1 as first-line chemotherapy.

**Table 2:** Patient characteristics (*n* = 6)

Variable	*N*	%
Age (years)		
Median	68	
Range	58–80	
Sex		
Male	6	100
Female	0	0
ECOG PS		
0	1	17
1	5	83
Primary tumor location		
Gastric	6	100
Gastro-esophageal junction	0	0
Histology		
Intestinal	6	100
Diffuse	0	0
Prior gastrectomy		
Yes	3	50
No	3	50
Number of metastatic sites		
Single	3	50
Multiple	3	50
Peritoneal metastasis		
Yes	1	17
No	5	83
Prior antineoplastic drugs		
Fluoropyrimidine	6	100
Platinum	5	83
Taxane	6	100
Trastuzumab	2	34
Ramucirumab	1	17
Prior number of regimens		
One	1	17
Two	4	67
Three	1	17

*ECOG PS* Eastern Cooperative Oncology Group performance status

### DLT

No DLT was observed at Level 1, and hence the RD was determined to be ramucirumab 8 mg/kg and irinotecan 150 mg/m^2^. No treatment-related deaths were observed.

### Toxicity and dose intensity

Toxicity was assessable in all patients. The worst adverse events through the protocol treatment period are listed in Table 3. Grade ≥ 3 neutropenia, anemia, and febrile neutropenia occurred in 17%, 17%, and 0% of patients, respectively. Grade ≥ 3 non-hematological toxicity occurred in only one patient (17%), namely hypertension, but this was treatable with an oral antihypertensive. No patients needed treatment delay of the second cycle. The median time to the first dose reduction of irinotecan was 2.5 cycles (range 2–3) in two of the six patients, due to neutropenia and anorexia, respectively. However, no patient needed treatment delay, discontinuation, or dose adjustment due to ramucirumab-related toxicities. The median percentage of relative dose intensity delivered during protocol treatment was 98.4% (range 55.5–112%) for irinotecan and 97.4% (range 60–112%) for ramucirumab.

**Table 3:** Maximum toxicity per patient during protocol treatment (*n* = 6)

Adverse event	NCI-CTC grade					
	1	2	3	4	All (%)	3/4 (%)
Hematologic						
Leukopenia	1	2	0	0	50	0
Neutropenia	2	1	1	0	67	17
Anemia	3	2	1	0	100	17
Thrombocytopenia	5	0	0	0	83	0
Alanine aminotransferase (ALT) increased	3	0	0	0	50	0
Aspartate aminotransferase (AST) increased	3	0	0	0	50	0
Increased bilirubin	0	1	0	0	17	0
Non-hematologic						
Anorexia	2	1	0	0	50	0
Alopecia	1	0	0	0	17	0
Constipation	2	0	0	0	33	0
Diarrhea	4	0	0	0	67	0
Dysgeusia	1	1	0	0	33	0
Edema	2	0	0	0	33	0
Epistaxis	2	0	0	0	33	0
Fatigue	2	1	0	0	50	0
Febrile neutropenia	–	–	0	0	0	0
Hoarseness	1	0	0	0	17	0
Hypertension	1	1	1	0	50	17
Myalgia	1	0	0	0	17	0
Nausea	2	0	0	0	33	0
Stomatitis	1	0	0	0	17	0
Thromboembolic event	0	1	0	0	17	0

*NCI-CTC* National Cancer Institute Common Toxicity Criteria

### Efficacy and treatment continuation

**Fig. 1:**
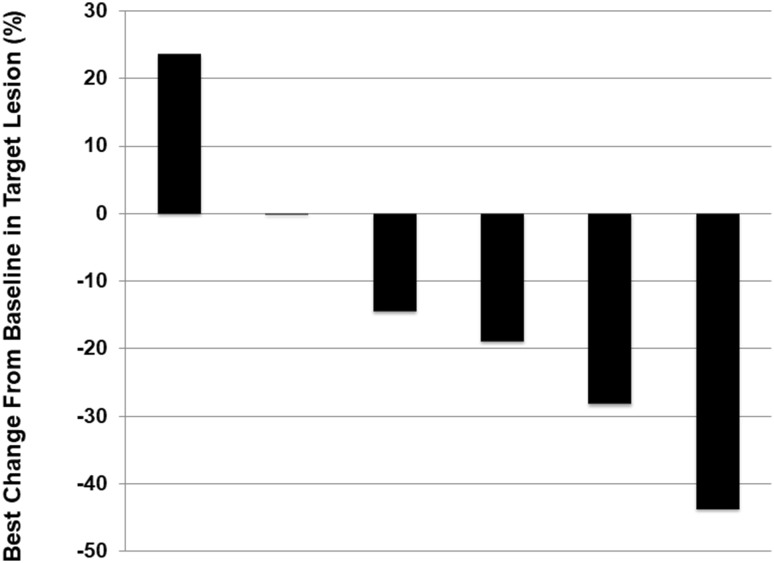
Maximum reduction in tumor burden from baseline in target lesions

Response was assessable in all six patients according to the RECIST criteria. Of the patients, one had a partial response, four had stable disease and one had progressive disease, giving a response rate (RR) of 17% and a disease control rate (DCR) of 83%, respectively. Maximum reduction in tumor burden from baseline in target lesions is shown Fig. 1. The greatest reduction in tumor burden was in a HER2-positive patient who had received combination chemotherapy consisting of capecitabine, cisplatin, and trastuzumab for first-line and paclitaxel plus trastuzumab as part of a second-line clinical trial, and then was enrolled this study. First-line chemotherapy with capecitabine, cisplatin, plus trastuzumab and second-line chemotherapy with paclitaxel plus trastuzumab had not shown antitumor effect at each first assessment; however, third-line chemotherapy with irinotecan plus ramucirumab showed outstanding antitumor effect for 7 months.

**Fig. 2:**
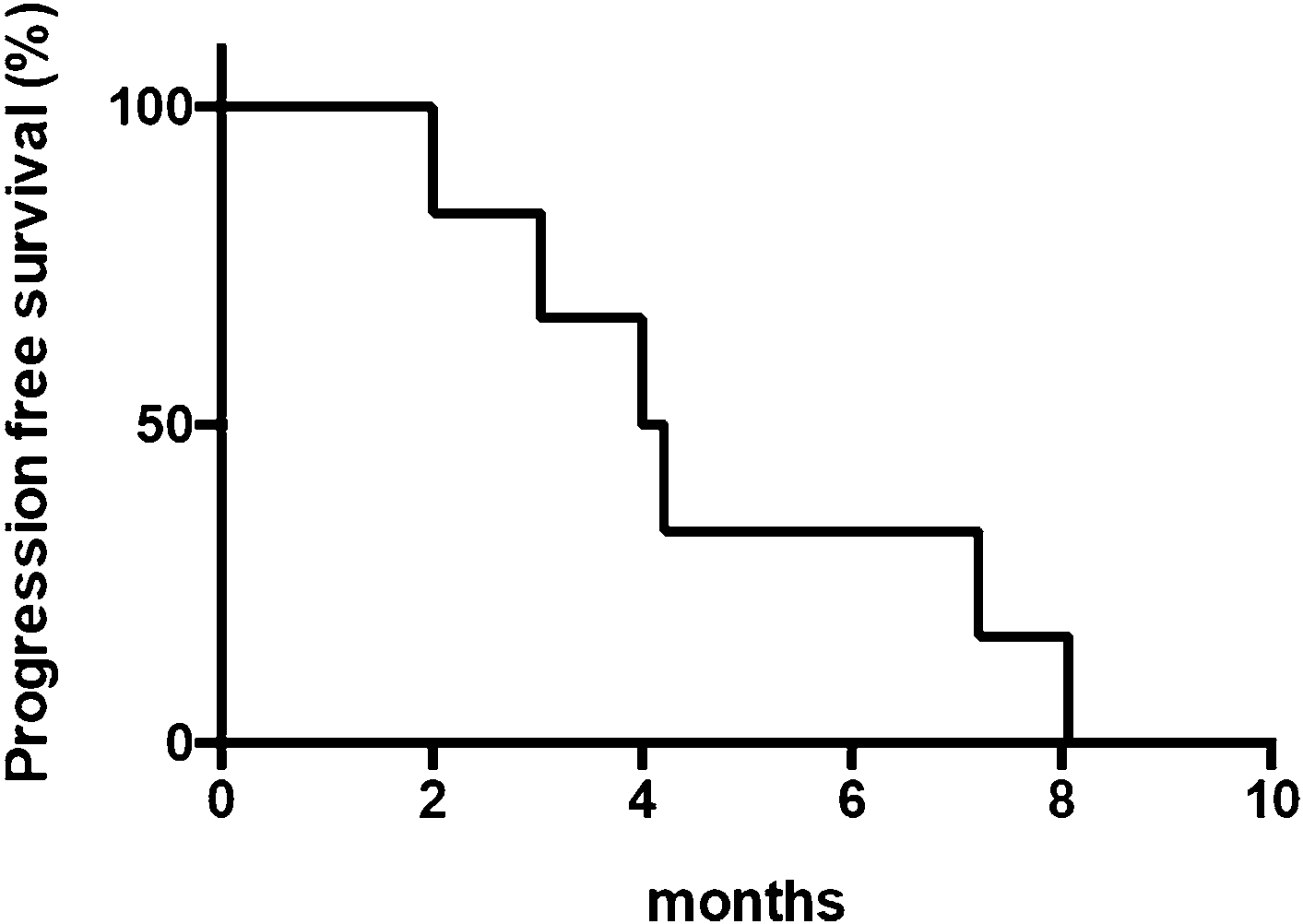
Length of progression-free survival (*n* = 6)

**Fig. 3:**
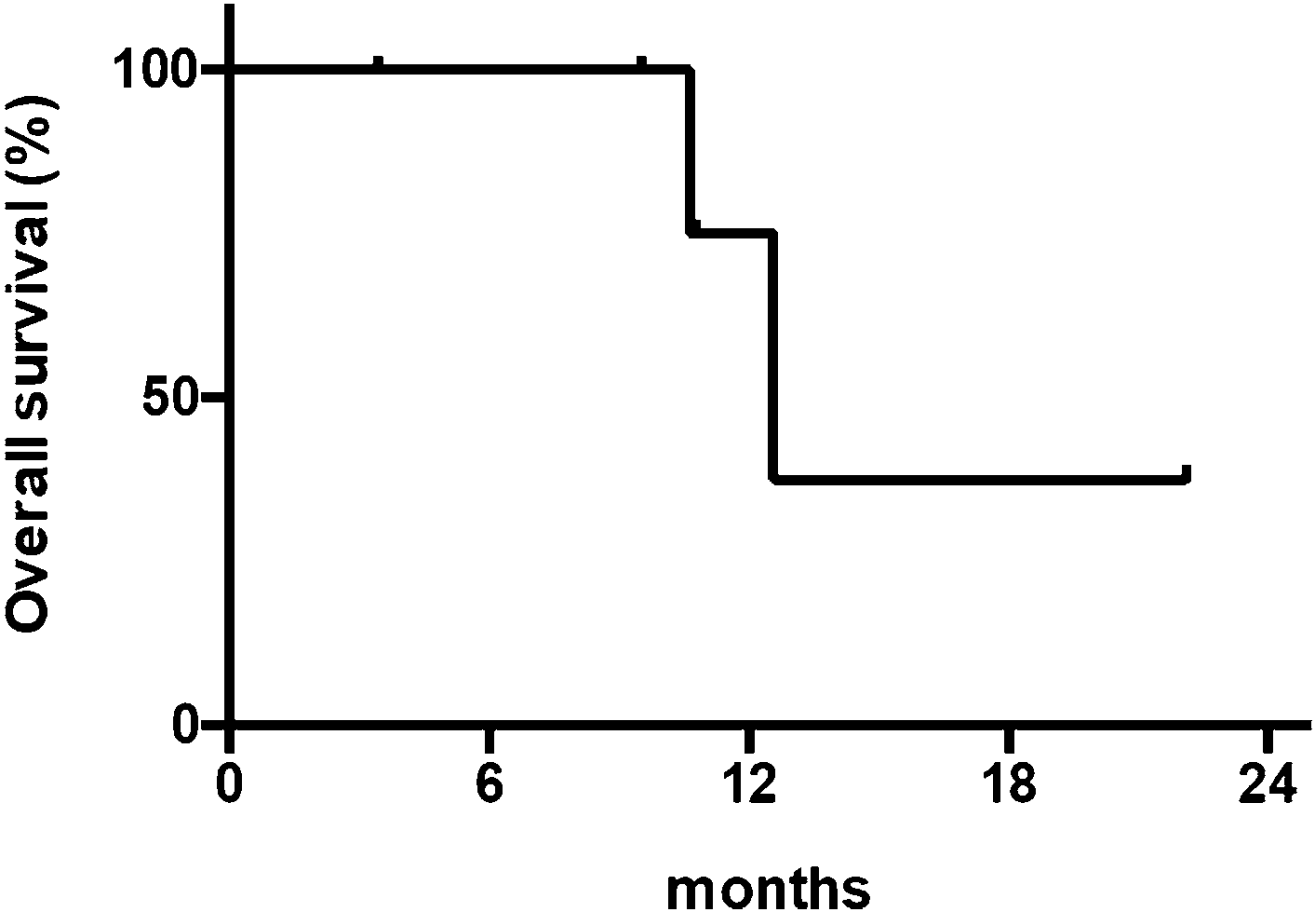
Length of overall survival (*n* = 6)

Of the six patients, all discontinued the protocol treatment due to disease progression. With a median follow-up of 10 months (range 3.4–22.1), the median PFS and OS was 4.0 months (95% CI 2.6–5.4) and 12.6 months (95% CI 9.7–15.5), respectively (Figs. 2, 3). Four of the six patients (67%) received subsequent chemotherapy after the protocol treatment.

## Discussion

This is the first report of the feasibility and activity of salvage chemotherapy consisting of irinotecan plus ramucirumab in patients with AGC. RDs of systemic chemotherapy with irinotecan plus ramucirumab were defined as irinotecan at 150 mg/m^2^ in combination with ramucirumab at 8 mg/kg.

Allowing for the small number of patients in this study, the safety of irinotecan plus ramucirumab appeared to be promising. Toxicities ≥ Grade 3 occurred in 17%, namely neutropenia, anemia, and hypertension, but all cases resolved without the use of G-CSF or transfusion. Two of the six patients needed dose reduction of irinotecan due to irinotecan-related toxicities (myelosuppression and gastrointestinal toxicity); however, no patient needed treatment delay, discontinuation or dose adjustment due to ramucirumab-related toxicities.

Nivolumab, a fully human IgG-4 monoclonal antibody inhibitor of programmed death-1 (PD-1), showed a survival benefit compared with placebo in patients with AGC at salvage-line setting [19]. In this randomized phase III trial, the nivolumab arm showed a median OS of 5.26 months, a median PFS of 1.61 months, an RR of 11.2%, and a DCR of 40.3%. Although efficacy was not the primary endpoint of this study, anti-tumor activity (RR 17%, DCR 83%) and survival benefit (OS 12.6 months, PFS 4.6 months) seem to be highly promising. These results suggest the efficacy of an irinotecan plus ramucirumab regimen in the salvage-line treatment of gastric cancer.

For VEGF inhibitors such as bevacizumab, preclinical and clinical studies in metastatic colorectal cancer (mCRC) suggest that there may be benefit in continuing treatment beyond progression [20], but it is still unknown whether AGC patients would benefit from such an approach. In this trial, one patient received ramucirumab plus paclitaxel as a second-line treatment and then enrolled in this study. The patient’s best response assessed after four cycles of the protocol treatment was disease progression.

A limitation related to the study design should be discussed. We planned a de-escalation design for the present study. The MTD for irinotecan is dependent on the disease status, PS and chemotherapy agents given in combination. In the clinical setting, AGC patients commonly receive irinotecan weekly (irinotecan monotherapy: 125 mg/m^2^ [21, 22]) or biweekly (irinotecan monotherapy: 150 mg/m^2^ [7, 23] or irinotecan 150–180 mg/m^2^ combined with fluorouracil and leucovorin [23–26]). In the present study, the dose of irinotecan did not reach the MTD. The question therefore remains whether irinotecan doses can be further increased in AGC patients without the UGT1A1*28/*28, *6/*6 or *28/*6 genotypes.

In conclusion, we found that systemic salvage chemotherapy with an irinotecan plus ramucirumab regimen was well-tolerated by patients with AGC. This phase I study demonstrates that the RDs for chemotherapy with irinotecan plus ramucirumab were irinotecan 150 mg/m^2^ in combination with ramucirumab 8 mg/kg. This regimen demonstrated sufficient activity to warrant further prospective study, and an intergroup phase III trial of Ramucirumab plus Irinotecan in third or more line Beyond progression after Ramucirumab for Advanced Gastric cancer is now ongoing as the RINDBeRG trial (no. UMIN000023065).
